# CT-based analysis of muscle volume and degeneration of gluteus medius in patients with unilateral hip osteoarthritis

**DOI:** 10.1186/s12891-017-1828-2

**Published:** 2017-11-15

**Authors:** Takako Momose, Yutaka Inaba, Hyonmin Choe, Naomi Kobayashi, Taro Tezuka, Tomoyuki Saito

**Affiliations:** Department of Orthopaedic Surgery, Yokohama City University, 3-9 Fukuura, Kanazawa-ku, Yokohama, Japan

**Keywords:** Gluteus medius, Muscle volume, Fatty degeneration, Cross-sectional area, Hip osteoarthritis

## Abstract

**Background:**

The gluteus medius (GMED) affects hip function as an abductor. We evaluated muscle volume and degeneration of the GMED by using CT-based analysis and assessed factors that affect hip abductor strength in patients with unilateral hip osteoarthritis (OA).

**Methods:**

We examined clinical and imaging findings associated with hip abductor strength in consecutive 50 patients with unilateral hip OA. Hip abductor muscle strength and Harris hip score (HHS) were assessed. Leg length discrepancy (LLD) and femoral offset were assessed using X-ray; CT assessment was employed for volumetric and qualitative GMED analysis. Volumetric analysis involved measurement of cross sectional area (CSA) and three-dimensional (3D) muscle volume. CT density was measured for the qualitative assessment of GMED degeneration with or without adjustment using a bone mineral reference phantom.

**Results:**

Hip abductor muscle strength on the affected side was significantly lower than that on the contralateral healthy side and positively correlated with overall score and score for limping of gait of HHS, demonstrating the importance of hip abductor strength for normal hip function. A significant correlation was found between CSA and 3D muscle volume, unadjusted CT density and adjusted CT density, and hip abductor strength and these CT measurements. Multiple linear regression analysis demonstrated that 3D muscle volume, adjusted CT density, and LLD are independent factors affecting hip abduction.

**Conclusions:**

3D measurement of muscle volume and adjusted CT density more accurately reflect quantity and the GMED quality than do conventional assessments. Increase in muscle volume, recovery of muscle degeneration, and correction of LLD are important for improving limping in patients with hip OA.

## Background

Hip abductor muscles are important determinants for hip function [1]. Abductor function ensures stability of the hip joint and controls pelvic posture in standing and walking [1]. The gluteus medius (GMED) is one of the main muscles in the hip abductor muscle group. The GMED provides stabilization of the pelvis in a single leg stance [1]. Thus, dysfunction of the GMED is responsible for unstable hip and postural imbalance of the pelvis during ambulation. A reduction in the volume of the GMED in patients with hip osteoarthritis (OA) is a major reason for limping gait [2–4]. Therefore, quantification of the GMED can provide vital information for obtaining normal gait in patients with hip OA.

Computed tomography (CT) is one of the commonly used imaging tools for the quantification of muscle volume around the hip [3, 5]. Measurement of cross-sectional area (CSA) has been demonstrated as a method for evaluating muscle volume [3, 5]; however, such measurements in cross-sectional images are widely variable and depend on the place of section. Since the GMED transverses from the pelvis to the femur and is widely variable in each patient [6], three-dimensional (3D) analysis can more precisely quantify GMED muscle volume. In addition to muscle volume, muscle quality is also responsible for muscle weakness [7, 8]. However, few studies have assessed the relationship between muscle quality and muscle strength around the hip, although measurement of CT density can be used to assess muscle degeneration [9]. CT density can be affected by CT scan settings; as demonstrated in measurements of bone mineral density, adjustment of CT density using a bone mineral reference phantom may improve the evaluation of muscle degeneration [10].

The purposes of our study were to evaluate muscle volume and muscle degeneration of GMED using CT based analysis, and to assess the effect of hip abductor muscle strength on hip function and factors affecting hip abductor strength in patients with unilateral hip OA. For this purpose, we quantified clinical parameters, X-ray findings, and CT measurements of the GMED. CT measurements involved muscle volume quantified by CSA and 3D reconstruction and muscle degeneration by CT density with or without adjustment using bone mineral reference phantom.

## Methods

### Patients

This prospective study was approved by our institutional review board. We enrolled 50 consecutive patients with unilateral hip OA between April 2012 and May 2014 with informed consent. Those patients suffered from symptomatic hip for an average of 9 years (range, 0.5-45 years) before initial presentation to our hospital. For clinical assessment, the Harris hip score (HHS) was recorded. To assess hip abductor strength, isometric hip abductor strength measurements were performed on all patients using a manual isokinetic/isometric dynamometer (microFET; Hoggan Health Industries, Inc., Draper, UT, USA). During the test, the participants lay on their side with the test leg up and with the dynamometer secured on the lateral side of their thigh. The pad on the dynamometer was centered over the distal lateral femur at a standardized point of 80% of the length between the greater trochanter and the lateral femoral condyle. The testing leg was positioned straight (0° of hip and knee flexion and 0° of hip rotation), whereas the non-testing leg was positioned in approximately 30° of hip flexion and 30° of knee flexion against the table for comfort and stability. During the hip abduction testing, a careful causation was given on the reproducibility of lower leg motion, especially in patients with severe hip pain. The participant was asked to perform three maximal isometric contractions for 5 s with 30 s of rest between the repetitions on each side. The peak force from the 3 trials was used for statistical analysis. Abductor muscle strength was measured by hip surgeons in our institution.

### Imaging tests

All patients were scanned with a plain anterior-posterior X-ray of the hip in a standing position and computed tomography (CT) using transaxial CT scans (Sensation16; Siemens AG, Erlangen, Germany). Scanner settings were approximately 120 kV and 300 mA, with a slice thickness of 1.5 mm. The 50 patients were examined in the supine position using a spiral CT-scanner with their pelvis in the neutral position and the lower limb placed in patient’s natural rotation. Natural rotation meant the lower leg position in relax position. Since stretch of muscle might affect the muscle volumes, we did not enforce lower legs to patella median position. Transverse images were obtained from the iliac crest to the condyles of the femur in each patient.

**Fig. 1:**
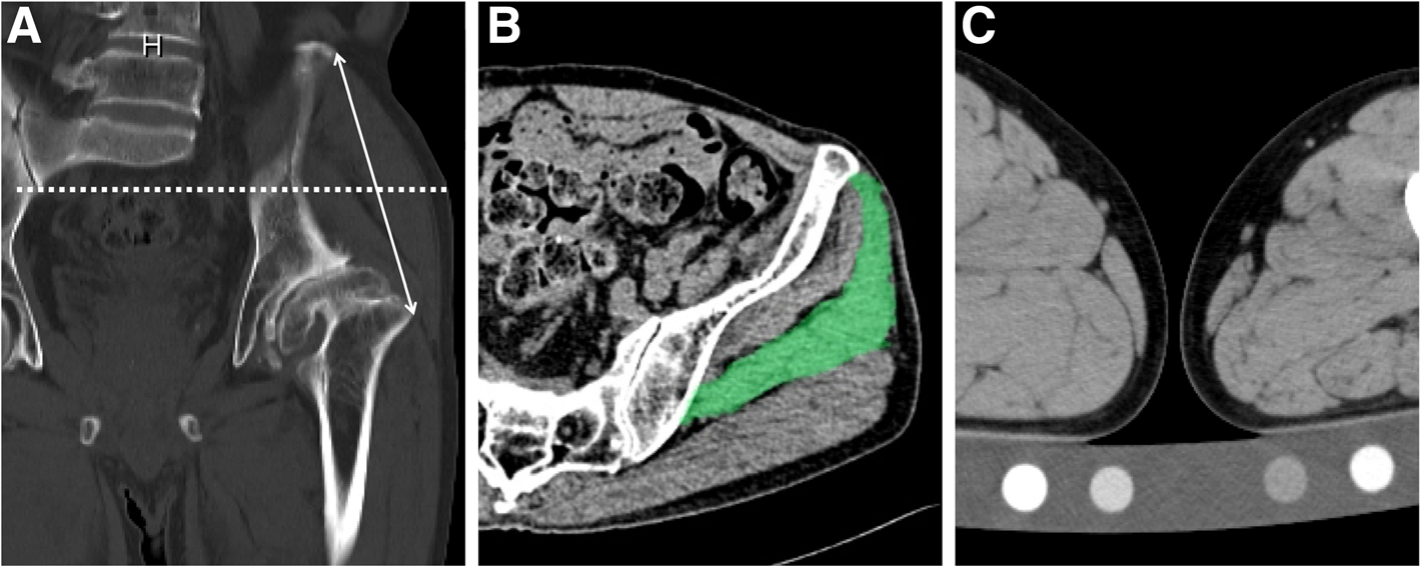
Cross-sectional analysis of the gluteus medius and bone mineral reference phantom for adjustment of CT density. **a** and **b** Cross sectional area (colored area in panel **b**) was measured using the mid-point of the anterior superior iliac spine and greater trochanter, which were visualized using cross-sectional images obtained by CT (dot line in panel **a**). **c** A calibration phantom (B-MAS 200; Kyoto-Kagaku, Kyoto, Japan) is placed in a CT scanner and an equivalent amount of hydroxyapatite in the bone mineral reference phantom was utilized to adjust CT density by using dedicated software

**Fig. 2:**
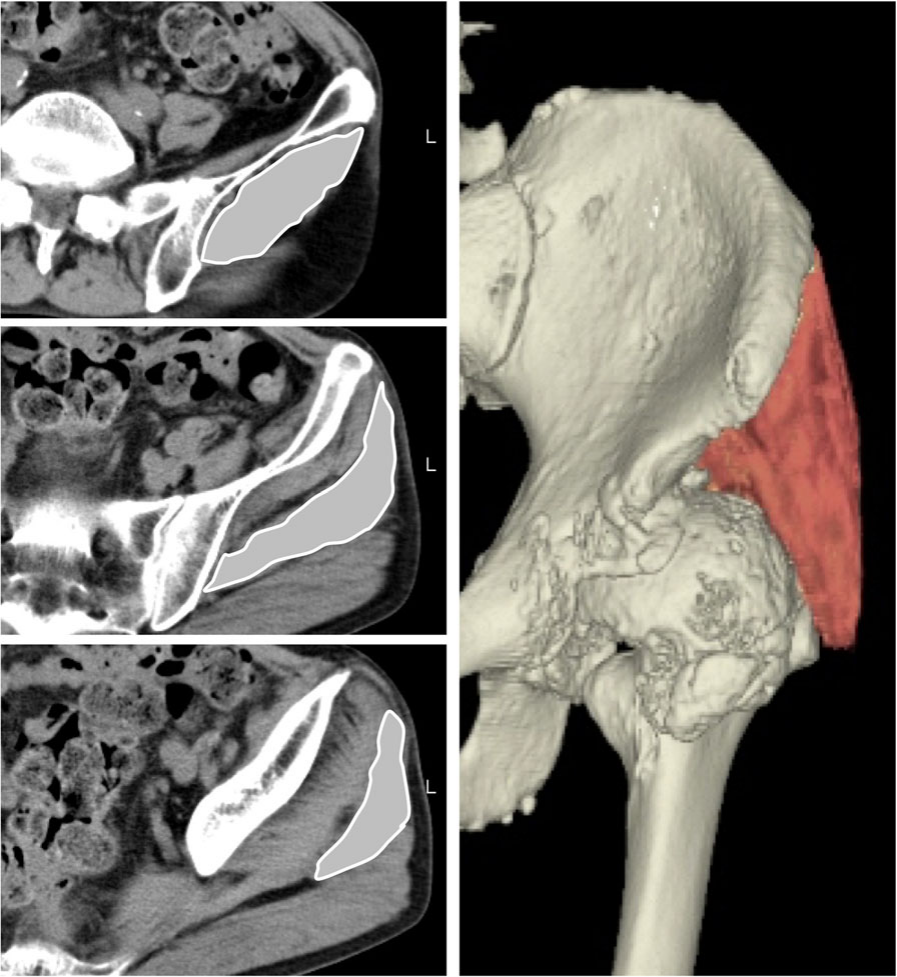
Muscle volume and fatty degeneration of the gluteus medius (GM). By tracing the cross-sectional areas of the GM on every third slice of the CT images using three-dimensional (3D) image analysis software (stripe area), GM muscle was reconstructed three-dimensionally (colored area). Muscle volume and fatty degeneration of the reconstructed GM were quantified using dedicated software

Leg length discrepancy [11] and femoral offset [12] and Kellgren and Lawrence (KL) grade [13] were measured using a plain anterior-posterior X-ray of the hip in a standing position. CSA was measured by locating the mid-point of the anterior superior iliac spine and greater trochanter using cross-sectional images obtained by CT (Fig. 1). Muscle volume was measured using dedicated software (Synapse Vincent®: Fujifilm Medical Systems, Japan). The software provided tools for measuring distances, areas, volumes, and radiological density. 3D muscle volume of the GMED was calculated in free-hand-draw fashion by tracking the margins of the GMED (Fig. 2). The margin of the GMED was drawn from proximal to distal slices, where the GMED was visible and on every third slice in between these start- and end-point slices. The decision to use every third slice was based on the minimum number of slices that the software required to trace the muscle margins and to calculate muscle volume. Whole muscle was carefully detected by excluding the surrounding fat or connective tissue and partial volume artifacts. The software reconstructed the GMED and output data included volume and CT density of the muscle (Fig. 2).

Muscle degeneration was quantified by calculating the mean CT density, which was measured as hounsfield units (HU). CT density was adjusted by calculating the equivalent hydroxyapatite amount in muscle area and a calibration phantom (B-MAS 200; Kyoto-Kagaku, Kyoto, Japan) using dedicated software that calculates adjusted HU of the muscle in the CT image based upon reference values measured in bone mineral reference phantom (Fig. 1) [14].

### Statistics

Pearson’s test was used for correlation analyses. Statistical significance of differences was determined by Student t-test or non-parametric Mann-Whitney test of data sets that were not normally distributed. To assess intra-observer and inter-observer reliabilities in measuring muscle volume of the GMED, intra-class correlation coefficients were calculated. For this purpose, 3D muscle volumes of the GMED in 12 patients were measured twice by the author with 6 months of interval period (TM) to determine intra-observer reliability, and those values in the same patients were also measured by another blinded orthopedic surgeon (HC) to determine inter-observer reliability. Multiple linear regression analysis was performed to identify the factors affecting abductor muscle strength. At first, a univariate regression analysis was performed with the following factors as explaining variables: muscle volume of the GMED, adjusted CT density, CSA of the GMED, CT density of the GMED, age, sex, BMI, LLD, femoral offset and KL grade. Secondly, all the variables were analyzed by multiple linear regression analysis (forced entry method). Thereafter, a backward selection method of multiple linear regression analysis was done with selected variables with *p* values less than 0.2 in forced entry method (final model). A *p*-value of <0.05 was considered significant.

## Results

Fifty patients (12 males and 38 females) with an average body mass index of 23.3 kg/m^2^ (standard deviation, ±4.1) and a mean age of 62 years (range, 30-82 years) were enrolled. Mean femoral offset on the affected hip was significantly lower than that on the healthy hip (34.6 mm and 38.3 mm, *p* < 0.01). Mean LLD was −10.9 mm (range, −55–0 mm) on the affected side and 4, 8 and 38 patients had a hip OA with KL grade 2, 3, and 4, respectively. The muscle strength of the hip abductor on the affected side was significantly lower than that on the contralateral healthy side (Table 1). The average ratio of the muscle strength of the hip abductor on the affected side to the healthy side was 69.9%. CSA and 3D muscle volume of the GMED on the affected side measured on CT images were significantly smaller than those on the contralateral healthy side in all patients (Table 1). The average ratios of the CSA and muscle volume on the affected side to those on the healthy side were 83.7% and 79.4%, respectively. CT density and adjusted CT density of the GMED on the affected side were significantly lower than those on the contralateral healthy side (Table 1). The average ratios of CT density and adjusted CT density of the GMED on the affected side to the healthy side were 78.2% and 76.5%, respectively. Hip abductor muscle strength had a positive correlation with overall HHS score and significantly correlated with the limping score, but not with the scores of hip joint pain, requirement of gait support, or walking distance in HHS (Table 2). Intra-observer reliability and inter-observer reliability for 3D muscle volume of the GMED were 0.998 (95% CI, 0.996–0.999) and 0.948 (95% CI, 0.887–0.977), respectively.

**Table 1:** Comparison of hip abductor strength and radiological measurements of the GMED between the affected side and healthy side

	Affected side (mean ± SD)	Healthy side (mean ± SD)	*P* value
Strength of hip abduction (N)	67.8 ± 31.0	94.5 ± 26.1	<0.001
Cross-sectional area of GMED (mm^2^)	1838 ± 494	2260 ± 503	<0.001
Muscle volume of GMED (cm^3^)	206 ± 57	260 ± 61	<0.001
CT density of the GMED (HU)	35.6 ± 10.6	45.5 ± 6.7	<0.001
Adjusted CT density of the GMED (mg/cm^3^)	29.1 ± 10.8	37.3 ± 7.2	<0.001

*GMED* gluteus medius, *HU* Hounsfield unit

**Table 2:** Correlation of hip abductor muscle on the affected side with Harris hip score (HHS)

HHS score (points)	r	*P* value
Overall	0.42	0.002
Pain	0.25	0.09
limping	0.51	<0.001
Required support for walking	0.11	0.45
Walking distance	0.22	0.13

To assess the factors that affect hip abductor muscle strength among clinical and radiological findings, univariate and multiple linear regression analysis were performed. Univariate linear regression analysis determined that CSA, 3D muscle volume, CT density, adjusted CT density, sex, and BMI, were factors that affected hip abductor muscle strength on the affected side. With further investigation using multiple linear regression analysis, 3D muscle volume, adjusted CT density, and LLD were found to be the factors that affected hip abductor strength on the affected side (Tables 3 and 4). Similar factors were found upon univariate analysis on the healthy side, but LLD was excluded from the factors affecting hip abductor strength in multivariate analysis.

**Table 3:** Univariate regression analysis for evaluating the association between hip abductor strength and clinical and radiological parameters on the affected side

	Regression coefficient	Standardized regression coefficient	*P* value
Muscle volume of the GMED	0.34	0.62	<0.001
Adjusted CT density of the GMED	1.36	0.47	0.001
CSA of the GMED	0.04	0.56	<0.001
CT density of the GMED	1.46	0.50	<0.001
Age	−0.04	−0.02	0.91
Sex	29.8	0.42	0.003
BMI	2.31	0.31	0.03
LLD	−0.17	−0.07	0.65
Femoral offset	0.32	0.06	0.68
KL grade	−0.01	0.02	0.31

*GMED* gluteus medius, *CSA* cross-sectional area, *RD* radiological density, *BMI* body mass index, *LLD* leg length discrepancy, *KL* Kellgren and Lawrence

**Table 4:** Multilinear regression analysis for evaluating the association between hip abductor strength and clinical and radiological parameters on the affected side

		Regression coefficient	Standardized regression coefficient	*P* value	Variance inflation factor
Forced Entry Method	Muscle volume of the GMED	0.28	0.52	0.07	6.63
	Adjusted CT density of the GMED	0.93	0.32	0.18	4.95
	CSA of the GMED	−0.003	−0.05	0.85	5.96
	CT density of the GMED	0.37	0.13	0.59	4.8
	Age	0.37	0.14	0.24	1.16
	Sex	−11.1	−0.15	0.35	2.44
	BMI	0.95	0.13	0.34	1.48
	LLD	0.71	0.28	0.09	2.31
	Femoral offset	0.42	0.08	0.55	1.49
	KL grade	−1.91	−0.04	0.77	1.46
Final model	Muscle volume of the GMED	0.3	.54	<0.001	1.29
	Adjusted CT density of the GMED	1.13	.39	0.005	1.65
	LLD	0.81	.32	0.014	1.43

*GMED* gluteus medius, *CSA* cross-sectional area, *RD* radiological density, *BMI* body mass index, *LLD* leg length discrepancy, *KL* Kellgren and Lawrence

**Fig. 3:**
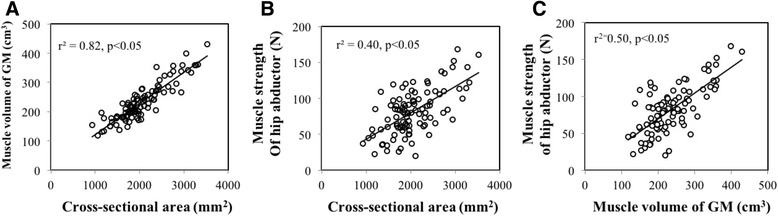
Correlation between cross-sectional area and muscle volume of the gluteus medius and muscle strength of hip abductor. **a** Cross-sectional area (CSA) and 3D muscle volume of the GMED were strongly correlated (r^2^ = 0.82, *p* < 0.05). **b** and **c** CSA and muscle volume measurements were both positively correlated with muscle strength of the hip abductor (r^2^ = 0.50, p < 0.05 and r^2^ = 0.40, p < 0.05, respectively)

**Fig. 4:**
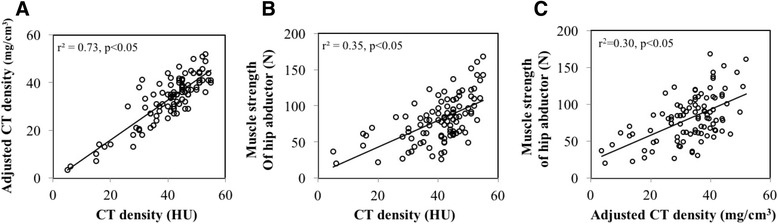
Correlation between CT density and adjusted CT density of the gluteus medius and muscle strength of the hip abductor. **a** CT density and adjusted CT density were significantly correlated (r^2^ = 0.73, p < 0.05). **b** and **c** CT density and adjusted CT density positively correlated with hip abductor strength (r^2^ = 0.30, p < 0.05 and r^2^ = 0.35, p < 0.05, respectively)

Cross-sectional and 3D muscle volume of the GMED showed a strong positive correlation (Fig. 3), and these measurements were both correlated with muscle strength of the hip abductor (Fig. 3). CT density and adjusted CT density showed a significant correlation (Fig. 4). Hip abductor strength correlated with CT density and adjusted CT density (Fig. 4). There was also a significant correlation between LLD and GMED volume (*r* = −0.19, *p* = 0.024) or LLD and adjusted CT density (*r* = −0.545, *p* < 0.001).

## Discussion

The purposes of our study were to evaluate the effect of hip abductor muscle strength on hip function, to validate the utility of our CT-based analysis for the quantification of muscle volume and muscle degeneration of GMED, and to assess factors that affect hip abductor strength in patients with unilateral hip OA. Reduction of muscle volume and degeneration of the GMED were evident in the present study, which agrees with findings of previous reports [2–4]. Correlation of hip abductor strength with limping score of HHS, rather than hip pain or walking distance, indicated the importance of hip abductor muscle strength for normal gait in patients with unilateral hip OA.

Previous studies have demonstrated an association between two-dimensional area of the GMED and hip abductor muscle strength [2–4]; however, few studies have focused on the 3D volume of the GMED. Measurements of the muscle CSA using MRI or CT imaging is a simple and easy way to determine muscle volume [2, 3, 5]. However, the shape of the muscle is widely variable, while cross-sectional analysis affects the place of the measurement section, particularly in patients with severe hip deformity. Because most patients with hip OA in Japan have acetabular dysplasia or femoral deformity, 3D quantification is attractive to assess muscle volume precisely. Therefore, in the present study, we performed 3D quantification of muscle volume and explored whether the 3D quantification is more likely associated with muscle strength. Although CSA has a strong correlation with 3D quantification of muscle volume, the results of multilinear regression analysis indicate that 3D measurement of muscle volume is a better method that accurately reflects muscle strength. The limitation of 3D quantification of muscle volume is that it requires the scanning of the entire muscle and additional time for 3D analysis. Assessment for muscle volume by cross-sectional images may be an alternative method for the quantification of muscle volume with minimal effort. However, intra-observer and inter-observer reliability analysis have demonstrated the good reproducibility of 3D measurement of muscle volume and we believe that 3D measurement is better to evaluate the accurate muscle volume of GMED in CT analysis.

Atrophic changes in muscle property may also be responsible for muscle weakness in patients with gait disorder. Nevertheless, quantification of muscle degeneration has not received sufficient attention. Upon CT assessment, several studies have measured radiological density to evaluate muscle degeneration [3, 5, 15]. However, radiological density can be affected by the different settings used during CT scanning. Hence, in the current study, we corrected CT values using bone mineral reference phantom for a more accurate assessment of muscle degeneration. Adjustment with bone mineral reference phantom is a widely used method to quantify bone mineral density using CT [14]. We applied this method to measure CT density in muscle area and investigated whether adjustment with bone mineral reference phantom improves the quantification of muscle properties in the GMED. In the current study, multilinear regression analysis indicated that adjusted radiological density of the GMED was a more important factor affecting hip abductor muscle strength compared to unadjusted CT density. The use of a bone mineral reference phantom eliminates the effect of different CT settings and ensures greater accuracy in assessing muscle degeneration using CT data.

No study yet has comprehensively demonstrated the effect of related factors on hip abductor muscle strength by using multilinear regression analysis. Few studies have evaluated the relevance of implant orientations, including hip center and femoral offset for hip abductor strength in total hip arthroplasty patients [12]. Patients with Hip OA have severe pain on the affected limbs, thereby disuse of abductor muscle caused muscle atrophy and reduction of volume in our patients. In addition to the 3D measurement of muscle volume and adjusted CT density, LLD, rather than femoral offset or KL grade, was found to be an important independent factor that affects abductor muscle strength in patients with unilateral hip OA. This finding indicates that LLD affects muscle strength in a different manner from muscle atrophic change, possibly because a superior shift in the hip center and greater trochanter may result in mechanical incompatibility of the hip abductor muscles on the affected side. Thus, correction of LLD can lead to improvement in hip abductor muscle properties and gait limping, as often observed in patients after total hip arthroplasty. The severity of OA quantified by KL grade on the affected side had no correlation to GMED strength, although the affected hip (KL grade 2-4) has significantly lower GMED strength than the healthy hip (KL grade 0) in this study. Because some patients in our series suffered from severe hip pain regardless of their KL grade, the severe pain of the affected hip likely eliminated the effect of the difference in KL grade on GMED strength.

One of the limitations in this study was that we only assessed the gluteus medius. Since hip motion requires complicated coordination of several muscles, assessment of other muscles is necessary for a practical understanding of functional disability in patients with hip OA. However, our volumetric and qualitative CT analysis may be useful in the assessment of other muscles and provides better information compared to CSA-based conventional analysis. The second limitation of this study was that we used a bone mineral reference phantom to adjust muscle density. The reference system, which was specifically prepared for muscle assessment, should be assessed in future CT-based studies. The third limitation was that discrepancies in strength testing may be related to hip pain, rather than true muscle weakness. During the testing, a careful causation was given on the reproducibility of lower leg motion, especially in patients with severe hip pain. However, although we tried to exclude the effect of hip pain on the testing, the severe hip pain might affect the measurement of muscle strength.

## Conclusions

In conclusion, we investigated factors that affected hip abductor muscle strength in patients with unilateral hip OA by using a novel volumetric and qualitative CT-based analysis of GMED. 3D measurement of muscle volume rather than CSA, adjusted CT density rather than unadjusted CT density, and LLD rather than femoral offset were found to be important factors that affect abductor muscle strength in patients with unilateral OA. An increase in muscle volume, recovery of muscle degeneration, and correction of LLD are important for improving gait limping in patients with hip OA.

## Abbreviations

3D: Three-dimensional
CSA: Cross sectional area
CT: Computed tomography
GMED: Gluteus medius
HHS: Harris hip score
HU: Hounsfield units
KL: Kellgren and Lawrence
LLD: Leg length discrepancy
OA: Osteoarthritis
